# Supplementary material for: Effect of community water fluoridation cessation on dental caries in 12-year-old Israeli children: a retrospective analysis of socioeconomic disparities (2014–2022)
Source: Isr J Health Policy Res. 2026 Sep 7;15:37. doi: 10.1186/s13584-026-00782-z (PMC13548607; doi:10.1186/s13584-026-00782-z)
Supplement: Supplementary file 1 [file 13584_2026_782_MOESM1_ESM.docx]

Effect of Community Water Fluoridation Cessation on Dental Caries in 12-Year-Old Israeli Children: A Retrospective Analysis of Socioeconomic Disparities (2014–2022)

Rayan Fawarsy^1^, Tali Chackartchi^2^, Mordechai Findler^3^, Haim Doron^4^, Jonathan Mann^1^, Guy Tobias^1^

Corresponding Author Guy Tobias

guy.tobias@mail.huji.ac.il

Abstract

Background

Israel’s community water fluoridation policy, mandatory from 2002 until its halt in 2014, provides a natural experiment to assess fluoridation’s impact on pediatric oral health. This retrospective study analyzed digital dental records of 12-year-old children (2014-2022) to assess changes in caries experience following community water fluoridation cessation.

Methods

We examined the Decayed, Missing, and Filled Teeth index, focusing on its Missing-and-Filled (MF) component, across socioeconomic status groups, gender, and fluoridation status (fluoridated vs non-fluoridated). Differences were assessed using one-way Welch ANOVA and post-hoc Games-Howell tests.

Results

The MF index increased significantly after fluoridation ended, especially in low-SES populations. Twelve-year-olds in low-SES communities without fluoridated water had more than double the caries experience of those in high-SES communities (mean MF 0.82 vs 0.40; p<0.05). Fluoridated communities showed a considerably narrower SES gap. SES had a large effect on caries outcomes (partial η²≈0.34), and the absence of fluoridation was associated with a modest but significant increase in MF (partial η²≈0.01). Females exhibited higher MF rates than males, with gender disparities more pronounced in non-fluoridated groups. Cessation of CWF was associated with a rise in caries and widened SES and gender disparities, underscoring fluoridation’s value in promoting oral health equity.

Conclusion

CWF remains a foundational public health measure, one that is unequaled in its ability to deliver effective, safe, equitable, and cost-saving caries prevention to entire populations.

Practical Implications

Reinstating mandatory community water fluoridation is essential to reverse the widening oral health disparities that disproportionately affect low-SES children and females. Policymakers must prioritize this measure to restore health equity and protect vulnerable populations who rely on this passive preventive intervention.

Keywords: Community water fluoridation, Public Health Dentistry, DMFT

Background

Dental caries (tooth decay) is one of the most common chronic diseases in children worldwide, caused by bacterial fermentation of dietary sugars leading to demineralization of tooth enamel^1^. If left untreated, caries can progress to pain, infection, and tooth loss, impacting quality of life and overall health^2,3,4^.

Fluoride is a well-established agent in caries prevention, and its use is a cornerstone of modern public health dentistry^5^. Fluoride’s caries-preventive effect occurs primarily through topical mechanisms: it enhances enamel remineralization and inhibits enamel demineralization in the presence of dietary acids, and it impairs the metabolism of cariogenic bacteria in dental plaque^6^. Over the past several decades, widespread exposure to fluoride (via toothpaste, rinses, varnishes, and drinking water) has led to marked improvements in child dental health globally^7^. Community water fluoridation (CWF), the adjustment of fluoride in public water supplies to optimal levels (around 0.7 ppm), is recognized as a safe, equitable, and cost-effective public health measure to reduce caries across populations^8^. International reviews indicate that water fluoridation is associated with a substantial reduction in caries experience in children (on the order of 25–30% fewer decayed teeth on average). Furthermore, evidence consistently shows that fluoride has a disproportionately beneficial effect for those most vulnerable to caries, such as children from lower socioeconomic groups, thereby helping to narrow oral health inequalities^9^.

In Israel, community water fluoridation was first implemented in 1981 and expanded nationwide in the early 2000’s. A 1998 law and subsequent regulations in 2002 made fluoridation mandatory in all large municipal water supplies, resulting in approximately 70–75% of the population receiving fluoridated water by 2013^10^. Studies documented that during the fluoridation era, Israeli children’s dental caries rates declined significantly, especially in communities with fluoridated water. However, in August 2014 the national fluoridation mandate was abruptly rescinded by the Ministry of Health under a new government, amid debates over individual choice and safety^8^. This policy change terminated fluoridation programs across Israel. Efforts to reinstate CWF were initiated in 2015–2016 by health authorities, citing the need to protect children’s oral health^11^. Nonetheless, bureaucratic and budgetary hurdles delayed re-fluoridation, and for several years most Israelis,including children nationwide, had no access to fluoridated drinking water^8,12^. This natural experiment raised concern that the gains in caries prevention might be lost, particularly among socioeconomically disadvantaged groups who have fewer resources to compensate for the lack of fluoridated water^13^.

Socioeconomic status is a known determinant of oral health. Even in high-income countries, children from lower SES families experience higher rates of dental caries and often have less access to dental care^14^. In Israel, national surveys demonstrated an inverse relationship between SES and caries: children in low-SES communities had significantly higher DMFT and untreated decay levels than those in affluent communities^15^. Fluoridation is thought to attenuate this gap by providing a baseline level of protection that does not depend on individual income or behavior. Indeed, research from other countries has shown that water fluoridation can reduce SES-based disparities in child dental caries. For example, in the U.K., a study comparing fluoridated Newcastle to unfluoridated Liverpool found that while lower-SES children had higher decay in both cities, the fluoridated community saw significantly less caries, particularly among the most deprived groups, thereby narrowing the inequality in dental decay rates^16^. Similarly, studies in Australia^17^ and South Korea^18^ have reported that fluoridation diminishes the influence of socioeconomic deprivation on caries. In fluoridated areas, children’s cavity rates tend to be low regardless of SES, whereas in non-fluoridated areas, there is a strong socioeconomic gradient (higher decay with lower SES). These findings suggest that the removal of fluoridation could exacerbate oral health inequities.

After Israel’s CWF cessation in 2014, initial data hinted at adverse trends. We (Tobias et al. 2022)^8^ analyzed national dental treatment rates for children ages 3–12 in Maccabi-Dent (Israel’s second-largest health fund dental service) and observed a significant increase in caries-related treatments in the years following fluoridation cessation. Notably, by 2016–2019 the number of restorative treatments needed was roughly double what it had been when fluoridation was in place. That study also found that children in low-SES localities experienced a greater rise in dental treatment needs than their high-SES peers, aligning with the concern that fluoridation withdrawal disproportionately harms disadvantaged groups. Another Israeli study^19^ reported a surge in severe dental infections: hospitalization rates for dental abscesses in children increased in nonfluoridated areas compared to optimally fluoridated areas, an effect most pronounced among low-SES populations. These observations underscore the potential consequences of the 2014 policy change on child oral health and health equity in Israel.

There remains a need for detailed analysis focusing on specific age cohorts and direct measures of caries experience following the fluoridation halt. The permanent first molars, which erupt around age 6-7, are key sentinel teeth for caries in school-age children, and by age 12 most children have all first molars erupted and potentially several years of exposure (or non-exposure) to fluoridated water on these teeth. By examining 12-year-olds, a standard index age for international caries comparisons^20^, we can assess the cumulative effect of fluoridation cessation on the caries that developed during the mixed dentition years.

The **DMFT index^21,22,23^** (Decayed, Missing, and Filled Teeth) is one of the most widely used epidemiological indicators for assessing **dental caries prevalence** in populations. It quantifies lifetime experience of caries by counting how many teeth are currently decayed (D), missing due to caries (M), or filled (F), providing a simple yet reliable measure of oral health status. Higher DMFT scores indicate poorer dental health and greater historical burden of tooth decay. In particular, evaluating the Missing and Filled (MF) component of the DMFT index in this age group provides insight into the portion of caries experience that resulted in treatment. This study focuses on the MF component because an increase in fillings and extractions over time may reflect new caries that have been addressed, presumably arising from insufficient prevention after fluoridation ended.

The aim of this study was to assess whether the 2014 cessation of CWF in Israel was associated with changes in dental caries among 12-year-old children, as measured by the MF component of the DMFT index, and whether these changes varied by socioeconomic status. We hypothesized that stopping fluoridation would increase caries, particularly in lower-SES communities, thereby widening existing oral health disparities. A secondary objective was to examine gender differences in caries outcomes, given evidence of differing caries patterns and dental service use between boys and girls. Analysis of data from 2014–2022 aimed to inform public health policy on the benefits of fluoridation and the need for equity-focused oral health interventions in Israel.

Methods

This retrospective study utilized electronic dental records from Maccabi-Dent, the dental division of Maccabi Healthcare Services (Israel’s second-largest HMO). The centralized database provided uniform data from over 50 clinics nationwide.

The study population comprised 12-year-old children treated between January 2014 and December 2022. This specific timeframe allows for a comparison between the tail end of national water fluoridation (2014) and the post-fluoridation era (2015–2022).

### Variables and Definitions

- Outcome Measure (MF Count): The primary outcome was dental caries experience, operationalized as the sum of restorative treatments (fillings/crowns) and extractions due to caries in permanent teeth. This corresponds to the "Filled" and "Missing" components of the DMFT index, indicating clinically significant caries requiring intervention.
- Socioeconomic Status (SES): Municipalities were classified into Low (clusters 1–3), Middle (4–6), and High (7–9) categories based on the Israel Central Bureau of Statistics 2015 locality index^24^.
- Fluoridation Exposure: Status (Yes/No) was assigned by linking the child's treatment record to their locality’s specific water fluoridation level for that year.
- Demographics: Gender and calendar year were included to assess utilization patterns and temporal trends.

### Ethics

All data were aggregated and de-identified to ensure privacy. The study was approved by the Maccabi Healthcare Services Institutional Review Board (Approval #MHS-0157-020).

Statistical Analysis

All analyses were conducted in SPSS (v27) and R (v4.1). We began with descriptive statistics, summarizing annual counts of MF-related treatments and mean MF values by SES, gender, and fluoridation status. To address the study objectives, we used **Welch’s one-way ANOVA**, which is robust to unequal variances and sample sizes, to compare MF scores by **SES** and **gender**, stratified by **fluoridation status**. This allowed us to evaluate SES–fluoridation and gender–fluoridation differences. We report Welch’s F, adjusted degrees of freedom, p-values, and partial η² as effect sizes. Significant findings were followed by **Games–Howell post-hoc tests**, with FDR correction (α=0.05). We also calculated **Cohen’s d** for key pairwise contrasts to quantify effect magnitude.All tests were two-tailed (p<0.05).

Results

From 2014 to 2022, Maccabi-Dent provided restorative or extraction services to approximately 28,000 distinct 12-year-old patients. Of the children receiving restorative treatments for caries (fillings or crowns), 55% were female and 45% male (27,998 total patients with at least one restoration: 15,400 girls, 12,598 boys). A smaller subset required extractions of permanent teeth due to caries: 1,545 patients in total (841 girls, 704 boys, ~54.4% female). In terms of the volume of procedures, 61,066 restorations and 3,327 caries-related extractions were recorded in the 12-year-old cohorts across 2014–2022. On average, when a child needed treatment, they received about 2.2 fillings and 2 extractions if extractions were needed, with similar distributions by gender (females had slightly more treatments per capita than males, consistent with their higher MF counts.

Socioeconomic distribution: The patient data encompassed a mix of localities spanning Israel’s SES spectrum. Approximately 30% of the 12-year-olds were from low SES communities, 45% from middle SES, and 25% from high SES.

### **MF Rate Analysis: Socioeconomic Status and Fluoridation (Fig. 1)**

We conducted two one-way Welch ANOVA tests to examine how socioeconomic status (SES) and fluoridation influence MF treatment rates. Both factors showed significant main effects:

- **SES:** MF rates differed significantly between high, middle, and low SES groups (F(2, 476.05)=242.34, p<0.05, η²=0.34).
- **Fluoridation:** The presence of fluoridated water also significantly affected MF rates (F(1, 400.51)=11.56, p<0.05, η²=0.009).

### **Post-hoc Findings (Games–Howell, FDR corrected)**

#### **Within Fluoridated Areas:**

- **High vs. Low SES:** High SES had significantly lower MF rates (0.361 vs. 0.660), large effect (d=1.436).
- **High vs. Mid SES:** High SES again had lower MF rates (0.361 vs. 0.572), large effect (d=1.046).
- **Low vs. Mid SES:** Difference not significant; effect small (d=0.374).

#### **Within Non-Fluoridated Areas:**

- **High vs. Low SES:** Large disparity (0.395 vs. 0.817), very large effect (d=1.935).
- **High vs. Mid SES:** High SES had lower MF rates (0.395 vs. 0.623), large effect (d=1.129).
- **Low vs. Mid SES:** Low SES had higher MF rates (0.817 vs. 0.623), large effect (d=0.801).

#### **Fluoridation Effect Within SES Groups:**

- **Low SES:** Higher MF rates without fluoridation (0.817 vs. 0.660), P<0.05.
- **Mid SES:** non-significant difference.
- **High SES:** non-significant difference.

FIG 1: Comparison between Socioeconomic Status and Fluoridation status

Gender Differences in Caries and the Role of Fluoride (FIG 2)

Welch’s ANOVA (one-way) comparing males vs females shows that gender had a significant main effect on the MF index (p<0.001). In the pooled data, 12-year-old girls averaged about 0.54 filled or missing teeth, whereas boys averaged 0.43, a statistically significant difference (~25% higher in females). The effect size for gender (partial η^2) was on the order of 0.01-0.02, indicating a small-to-moderate effect, but given the large sample this difference is meaningful in absolute terms. We further examined gender differences within the fluoridated and non-fluoridated groups to see if the gap changed with fluoride exposure.

In communities with fluoridation, girls had an average MF rate of 0.546, compared to 0.429 for boys. This difference was statistically significant (Games-owell p<0.05). In communities without fluoridation, the gender gap was even more pronounced: the analysis indicates that females in non-fluoridated localities had a significantly higher MF than males, with the difference larger than in the fluoridated scenario (p<0.01).

Figure 2 show that both girls and boys experienced an increase in caries after fluoridation ended, but girls had a higher caries burden than boys at baseline and their condition worsened relatively more without fluoride. Our post-hoc comparisons found: (a) With fluoride, the girl-boy difference was significant (Δ≈0.117 MF); (b) Without fluoride, the girl- boy difference was also significant and larger (Games–Howell test confirming a greater Δ); (c) For girls, being in a non-fluoridated area was associated with a higher MF compared to girls with fluoride (difference indicating fluoridation benefit for girls); (d) For boys, the MF was also higher without fluoride than with, though the relative increase was smaller than in girls. All these comparisons were statistically significant except the boy fluoride vs no-fluoride difference was marginally significant (small effect).

In fluoridated areas, girls had about 27% more caries-related treatments than boys (0.546 vs 0.429). The percentage increase in MF due to fluoridation cessation was roughly 20% for girls versus ~10% for boys, indicating that girls benefited more from fluoride and were more adversely affected by its removal.

FIG 2: Gender Differences in Caries and the Role of Fluoride

Discussion

This retrospective epidemiological study provides compelling empirical evidence regarding the public health consequences of the 2014 cessation of mandatory community water fluoridation in Israel. By analyzing national data on 12-year-old children over a period of eight spanning non-fluoridated years, our findings indicate a detrimental impact on dental health, characterized by a statistically significant rise in the mean Missing and Filled index following the policy reversal. This measurable increase in treated caries signals a regression in national oral health achievements. More critically, our analysis reveals that the burden of this policy shift has not been borne equally. The cessation of CWF has disproportionately affected children from lower socioeconomic status backgrounds and has exacerbated disparities between genders, effectively dismantling a structural intervention that had previously operated as a powerful social equalizer compared to downstream behavioral interventions^25^.

Perhaps the most profound implication of our findings lies in the realm of health equity. Prior to 2014, Israel, like many developed nations, struggled with substantial oral health inequalities, where children from disadvantaged communities carried a heavier burden of dental decay. During the era of mandatory fluoridation, however, these inequalities were significantly moderated. Our data indicates that in localities benefiting from CWF, the differential in caries experience between low- and middle-SES children was minimal, and the gap between the highest and lowest SES strata, while present, was markedly narrower than in the post-cessation period^8,15^.

This phenomenon aligns strongly with the "inverse equity hypothesis" in public health. Originated by Victora et al., this hypothesis posits that new public health interventions, particularly those requiring individual behavioral change, initially benefit higher socioeconomic groups who have better access to information and resources, thereby increasing inequalities^26^. Conversely, universal "upstream" preventive measures that require minimal individual effort such as CWF, tend to reduce health disparities by ensuring baseline protection for vulnerable populations who may lack access to other preventive modalities like regular professional dental care or high-quality fluoridated dentifrices^25,27^.

The 2014 policy change transformed Israel into a natural experiment in removing a fundamental layer of health protection. The result, as our stratified data demonstrate, has been a sharp regression toward a more unequal distribution of disease. In the absence of systemic fluoridation, the protective layer for disadvantaged children collapsed. Low-SES children now experience significantly higher rates of caries compared to their wealthy peers, a gap that is wider now than when fluoridation was in place. This suggests that affluent families possess the resources, knowledge, and access to compensate for the loss of fluoridated water through alternative means, whereas disadvantaged families do not.

Gender Disparities and Biological Vulnerability

Our study also highlighted consistent differences in caries experience by gender, adding another layer of complexity to the impact of cessation. We observed that across the study period, 12-year-old females exhibited consistently higher MF indices than their male counterparts. While gender gaps in oral health are generally less pronounced than SES gaps, this finding aligns with extensive international research^28^. The etiology of this difference is multifactorial. Biologically, girls typically experience earlier tooth eruption than boys; consequently, their permanent teeth are exposed to the oral environment and potential cariogenic challenges due to dietary habits or hormonal fluctuations affecting saliva for a longer duration by age 12^29^. Behaviorally, a higher propensity among females to utilize dental services may also result in more treated caries being captured by the MF index versus untreated decay in males.

Crucially, our interaction analysis suggests that female children may have derived slightly greater protective benefit from CWF when it was active. If early-erupting teeth in girls are inherently at higher risk due to longer post-eruptive exposure times, the presence of systemic fluoride during those critical years would provide disproportionate benefit. Consequently, the removal of fluoridation appears to have exacerbated gender disparities, as girls potentially regressed in the absence of the protective factor. While gender is rarely the primary driver of oral health policy, this finding suggests that preventive programs in a non-fluoridated landscape may need to be sensitive to biological and behavioral differences between sexes to ensure equitable outcomes.

The Israeli Context as a Red flag

Israel’s experience provides risk assessment and mitigation for policymakers globally regarding the consequences of reversing established evidence-based public health interventions. The 2014 decision to halt CWF was driven largely by political considerations and arguments framing fluoridation as an infringement on individual liberty, despite vociferous warnings from the dental public health community, both within Israel and internationally, that such a move would harm the most vulnerable populations^30^. Our findings validate those warnings. Within a relatively short span of 5–8 years following cessation, the caries trend reversed, confirming that the previous CWF program had been actively preventing a significant burden of disease.

Furthermore, this increase carries substantial economic implications. The rise in the MF index translates directly to increased treatments: restorations, endodontic procedures and extractions, placing a greater financial strain on both the public healthcare system that subsidizes pediatric dental care and on families incurring out-of-pocket expenses. This echoes findings from other jurisdictions that ceased fluoridation, such as Calgary, Canada, where researchers documented rising caries rates in primary teeth following cessation, prompting renewed debates on reinstatement due to both health and economic costs^31^. Systematic reviews consistently demonstrate that CWF is cost-saving, particularly in communities with high caries risk^32^.

Ethical Considerations for Policy

The disproportionate suffering of children from socioeconomically weaker sectors raises a fundamental ethical issue for health policy. A primary goal of public health policy, as articulated in frameworks by the Nuffield Council on Bioethics, should be the reduction of avoidable inequalities and the protection of vulnerable populations who cannot adequately protect themselves^33^. In this instance, a deliberate policy change actively increased inequality.

Ethical and Health Equity Implications of Discontinuing Community Water Fluoridation.

Discontinuing community water fluoridation should not be regarded as a neutral policy change, but as the withdrawal of an established population-wide preventive intervention supported by a substantial scientific evidence base. Contemporary population studies continue to associate fluoridation with a lower prevalence of severe childhood caries and fewer preventable dental hospitalizations, with some of the greatest benefits observed in socioeconomically deprived communities^34,35^. Moreover, community water fluoridation provides protection independently of individual income, health literacy, health behavior, or access to dental services, whereas alternatives such as fluoride toothpaste, professional fluoride application, and regular dental care require individual resources and sustained engagement^36^. Thus, withdrawing fluoridation may transfer responsibility for caries prevention from the state to families and disproportionately burden those already experiencing poorer oral health and limited access to preventive care, thereby widening existing inequalities. Although respect for autonomy and concerns regarding involuntary exposure warrant consideration, public health ethics requires these concerns to be balanced against beneficence, non-maleficence, distributive justice, proportionality, and the collective health benefit of the intervention^37,38^. Accordingly, terminating fluoridation can be ethically justified only following a transparent benefit- risk and health-equity assessment, meaningful public participation, implementation of adequately funded and universally accessible alternatives before cessation, and continued surveillance of caries and socioeconomic disparities after the policy change.

Our study provides critical, localized data reinforcing the urgency of full reinstatement. The magnitude of the differences observed provides compelling evidence for policymakers that CWF is not merely a clinical option but a moral imperative for health justice.

Limitations

This study’s strength lies in its use of a large, nationwide administrative dataset generated through a uniform recording system across more than 50 dental clinics. Nevertheless, several limitations should be acknowledged. First, the database was structured according to treatment rather than tooth-level diagnostic codes; therefore, untreated decay (the D component of DMFT) could not be reliably identified. The outcome was consequently operationalized as an MF treatment index, reflecting caries that resulted in restoration or extraction rather than total caries prevalence. The F component provides a pragmatic proxy for treated caries burden and restorative dental treatment need: among approximately 28,000 children, restorations accounted for 61,066 procedures, compared with 3,327 caries-related extractions. Thus, the findings were driven predominantly by restorative treatment. Although this measure may underestimate untreated disease and may be influenced by dental-service utilization, its large scale and consistent coding support internal comparisons between study groups. Second, fluoride exposure was assigned ecologically by locality and calendar year rather than individual residential history or water consumption; migration may therefore have introduced exposure misclassification. Finally, focusing on 12-year-olds, although consistent with international surveillance standards, limits generalizability to younger children whose primary dentition developed entirely after fluoridation ceased.

Recommendations and Future Directions

Based on these findings, we strongly recommend the following actions:

1. Urgent Reinstatement of CWF: Israeli health authorities must prioritize the rapid and complete reinstatement of CWF in all major water supplies, with particular urgency for systems serving lower-SES communities. This should be treated as a national infrastructure priority, supported by dedicated central government funding to overcome municipal budgetary hurdles.
2. Targeted Interim Preventive Measures: Recognizing that full reinstatement takes time, intensified interim preventive strategies must be deployed immediately in non-fluoridated, low-SES areas. This includes expanded school-based fluoride varnish programs, subsidized distribution of high-fluoride dentifrices, and targeted oral health education.
3. Enhanced Surveillance: The post-2014 era underscores the critical need for continuous national oral health surveillance. We recommend regular epidemiological surveys including younger age groups (e.g., age 5–6) and assessing untreated decay to monitor trends and evaluate the effectiveness of re-fluoridation efforts as they come online.

Future research should focus on a "reversal of the reversal" analysis, examining whether caries rates begin to decline again in municipalities that have resumed fluoridation.

Conclusion

CWF remains a foundational public health measure, one that is unequaled in its ability to deliver effective, safe, equitable, and cost-saving caries prevention to entire populations. The reinstatement of fluoridation in Israel is an urgent public health priority to ensure a healthier and more equitable future for all children.

List of abbreviations

DMFT- Decay Missing Filled Tooth

MFT- Missing Filled Tooth

SES- Socio Economic Status

CWF- community water fluoridation

Declarations

Ethical approval- The present study was approved by the Institutional Review Board (IRB) MHS-0157-20 in accordance with the Declaration of Helsinki byThe Helsinki committee of Maccabi Healthcare Services.

Availability of data and materials- upon request

Competing interests- The authors declare that they have no competing interests.

Funding- Funding was provided by MaccabiDent Dental health services.

Consent to Publish declaration: not applicable

Human Ethics and Consent to Participate declarations: not applicable’

Authors’ Contribution

TG- Conception and design of the study, acquisition of data, analysis and interpretation of data and drafting the article. FM- Conception and design of the study, interpretation of data and drafting the article. CT- Conception and design of the study, interpretation of data and drafting the article. RF- Conception and design of the study, interpretation of data and drafting the article. JM- Interpretation of data. HD- Conception and design of the study. All authors read and approved the final manuscript.

References

1. Spatafora G, Li Y, He X, Cowan A, Tanner ACR. The Evolving Microbiome of Dental Caries. Microorganisms. 2024 Jan 7;12(1):121. doi: 10.3390/microorganisms12010121. PMID: 38257948; PMCID: PMC10819217.
2. Souza JGS, Souza SE, Noronha MDS, Ferreira EFE, Martins AMEBL. Impact of untreated dental caries on the daily activities of children. J Public Health Dent. 2018 Jun;78(3):197-202. doi: 10.1111/jphd.12259. Epub 2017 Nov 29. PMID: 29193108.
3. Abdel Fattah MA, Barghouth MH, Wassel MO, Deraz OH, Khalil AE, Sarsik HM, Mohsen AMA, Qenawy AS, Abou El Fadl RK. Epidemiology of dental caries in permanent dentition: evidence from a population-based survey in Egypt. BMC Public Health. 2022 Dec 27;22(1):2438. doi: 10.1186/s12889-022-14844-9. PMID: 36575430; PMCID: PMC9794108.
4. Broadbent JM, Thomson WM. For debate: problems with the DMF index pertinent to dental caries data analysis. Community Dent Oral Epidemiol. 2005 Dec;33(6):400-9. doi: 10.1111/j.1600-0528.2005.00259.x. PMID: 16262607; PMCID: PMC1388190.
5. Veneri F, Vinceti SR, Filippini T. Fluoride and caries prevention: a scoping review of public health policies. Ann Ig. 2024 May-Jun;36(3):270-280. doi: 10.7416/ai.2024.2593. Epub 2024 Jan 17. PMID: 38236001.
6. Pollick H. The Role of Fluoride in the Prevention of Tooth Decay. Pediatr Clin North Am. 2018 Oct;65(5):923-940. doi: 10.1016/j.pcl.2018.05.014. PMID: 30213354.
7. Iheozor-Ejiofor Z, Walsh T, Lewis SR, Riley P, Boyers D, Clarkson JE, Worthington HV, Glenny AM, O'Malley L. Water fluoridation for the prevention of dental caries. Cochrane Database Syst Rev. 2024 Oct 4;10(10):CD010856. doi: 10.1002/14651858.CD010856.pub3. PMID: 39362658; PMCID: PMC11449566.
8. Tobias G, Mordechai F, Tali C, Yaron B, Beatrice GP, Jonathan M, Harold SC. The effect of community water fluoridation cessation on children's dental health: a national experience. Isr J Health Policy Res. 2022 Jan 28;11(1):4. doi: 10.1186/s13584-022-00514-z. PMID: 35090561; PMCID: PMC8796457.
9. Centers for Disease Control and Prevention (CDC). Populations receiving optimally fluoridated public drinking water--United States, 1992-2006. MMWR Morb Mortal Wkly Rep. 2008 Jul 11;57(27):737-41. PMID: 18614991.
10. Vered Y, Sgan-Cohen H. [The fight over water fluoridation in Israel]. Refuat Hapeh Vehashinayim (1993). 2002 Apr;19(2):19-23, 76. Hebrew. PMID: 12055706.
11. <https://www.timesofisrael.com/deputy-health-minister-moves-to-restore-water-fluoridation/#:~:text=Deputy%20Health%20Minister%20Yaakov%20Litzman,year%2C%20Army%20Radio%20reported%20Sunday> (11\25)
12. Shahrabani S, Benzion U, Machnes Y, Gal A. The use of dental services for children: implications of the 2010 dental reform in Israel. Health Policy. 2015 Feb;119(2):117-26. doi: 10.1016/j.healthpol.2014.11.007. Epub 2014 Nov 15. PMID: 25465981.
13. Sgan-Cohen H, Tobias G, Zini A. Is the oral health reform in Israel optimally distributed? - A commentary. Isr J Health Policy Res. 2019 Mar 20;8(1):33. doi: 10.1186/s13584-019-0302-z. PMID: 30894215; PMCID: PMC6425632.
14. Wong E. The real cost of removing water fluoridation. A health equity impact assessment. Wellesley Institute; 2013. [www.wellesleyinstitute.com](http://www.wellesleyinstitute.com).
15. Tobias G, Khaimov A, Zini A, Sgan-Cohen HD, Mann J, Chotiner Bar-Yehuda Y, Aflalo E, Vered Y. Caries prevalence and water fluoridation in Israel: a cross-sectional study. Quintessence Int. 2024 Feb 28;55(2):166-172. doi: 10.3290/j.qi.b5003045. Epub 2023 Dec 21. PMID: 38414368.
16. Jones CM, Worthington H. Water fluoridation, poverty and tooth decay in 12-year-old children. J Dent. 2000 Aug;28(6):389-93. doi: 10.1016/s0300-5712(00)00005-1. PMID: 10856802.
17. Australian Research Centre for Population Oral Health, The University of Adelaide, South Australia. The benefits of water fluoridation across areas of differing socio-economic status. Aust Dent J. 2008 Jun;53(2):180-3. doi: 10.1111/j.1834-7819.2008.00030.x. PMID: 18494976.
18. Cho HJ, Lee HS, Paik DI, Bae KH. Association of dental caries with socioeconomic status in relation to different water fluoridation levels. Community Dent Oral Epidemiol. 2014 Dec;42(6):536-42. doi: 10.1111/cdoe.12110. Epub 2014 May 30. PMID: 24890821.
19. Klivitsky A, Tasher D, Stein M, Gavron E, Somekh E. Hospitalizations for dental infections: optimally versus nonoptimally fluoridated areas in Israel. J Am Dent Assoc. 2015 Mar;146(3):179-83. doi: 10.1016/j.adaj.2014.12.011. PMID: 25726345.
20. Grieshaber A, Waltimo T, Haschemi AA, Bornstein MM, Kulik EM. Dental caries and associated factors in 7-, 12- and 15-year-old schoolchildren in the canton of Basel-Landschaft, Switzerland: Changes in caries experience from 1992 to 2021. Int J Paediatr Dent. 2024 Mar;34(2):169-178. doi: 10.1111/ipd.13122. Epub 2023 Oct 9. PMID: 37807838.
21. Oral health surveys: basic methods - 5th edition (2013) <https://www.who.int/publications/i/item/9789241548649>
22. Dumitrescu R, Sava-Rosianu R, Jumanca D, Balean O, Damian LR, Fratila AD, Maricutoiu L, Hajdu AI, Focht R, Dumitrache MA, Daguci C, Postolache M, Vernic C, Galuscan A. The Impact of Parental Education on Schoolchildren's Oral Health-A Multicenter Cross-Sectional Study in Romania. Int J Environ Res Public Health. 2022 Sep 5;19(17):11102. doi: 10.3390/ijerph191711102. PMID: 36078817; PMCID: PMC9518154.
23. Vakani F, Basaria N, Katpar S. Oral hygiene KAP assessment and DMFT scoring among children aged 11-12 years in an urban school of Karachi. J Coll Physicians Surg Pak. 2011 Apr;21(4):223-6. Erratum in: J Coll Physicians Surg Pak. 2011 May;21(5):322. PMID: 21453619.
24. Central Bureau of Statistics(2022) <https://www.cbs.gov.il/he/publications/DocLib/isr_in_n/sr_in_n22e.pdf>
25. Watt RG. Strategies and approaches in oral disease prevention and health promotion. Bull World Health Organ. 2005 Sep;83(9):711-8. Epub 2005 Sep 30. PMID: 16211164; PMCID: PMC2626336.
26. Victora CG, Vaughan JP, Barros FC, Silva AC, Tomasi E. Explaining trends in inequities: evidence from Brazilian child health studies. Lancet. 2000 Sep 23;356(9235):1093-8. doi: 10.1016/S0140-6736(00)02741-0. PMID: 11009159.
27. McLaren L, Patterson S, Thawer S, Faris P, McNeil D, Potestio M. Fluoridation cessation: More science from Alberta. Community Dent Oral Epidemiol. 2017 Dec;45(6):503-505. doi: 10.1111/cdoe.12346. Epub 2017 Oct 10. PMID: 28994455; PMCID: PMC5698747.
28. Lukacs JR, Largaespada LL. Explaining sex differences in dental caries prevalence: saliva, hormones, and "life-history" etiologies. Am J Hum Biol. 2006 Jul-Aug;18(4):540-55. doi: 10.1002/ajhb.20530. PMID: 16788889.
29. Ferraro M, Vieira AR. Explaining gender differences in caries: a multifactorial approach to a multifactorial disease. Int J Dent. 2010;2010:649643. doi: 10.1155/2010/649643. Epub 2010 Mar 16. PMID: 20339488; PMCID: PMC2840374.
30. Zusman, S.P. Water Fluoridation in Israel: Ethical and Legal Aspects. Public Health Rev 34, 6 (2012). <https://doi.org/10.1007/BF03391658>
31. McLaren L, Patterson S, Thawer S, Faris P, McNeil D, Potestio M, Shwart L. Measuring the short-term impact of fluoridation cessation on dental caries in Grade 2 children using tooth surface indices. Community Dent Oral Epidemiol. 2016 Jun;44(3):274-82. doi: 10.1111/cdoe.12215. Epub 2016 Feb 17. PMID: 26888380; PMCID: PMC5021129.
32. Mariño R, Zaror C. Economic evaluations in water-fluoridation: a scoping review. BMC Oral Health. 2020 Apr 16;20(1):115. doi: 10.1186/s12903-020-01100-y. PMID: 32299417; PMCID: PMC7164347.
33. Nuffield Council on Bioethics. Public health: ethical issues. London: Nuffield Council on Bioethics; 2007. <https://www.nuffieldbioethics.org/publication/public-health-ethical-issues/> (accessed 11/25)
34. Hobbs, M., Wade, A., Jones, P., Marek, L., Tomintz, M., Sharma, K., McCarthy, J., Mattingley, B., Campbell, M., & Kingham, S. (2020). Area-level deprivation, childhood dental ambulatory sensitive hospitalizations and community water fluoridation: Evidence from New Zealand. International Journal of Epidemiology, 49(3), 908–916. [PubMed PMID: 32347945](https://pubmed.ncbi.nlm.nih.gov/32347945/?utm_source=chatgpt.com)
35. Schluter, P. J., Hobbs, M., Atkins, H., Mattingley, B., & Lee, M. (2020). Association between community water fluoridation and severe dental caries experience in 4-year-old New Zealand children. JAMA Pediatrics, 174(10), 969–976. [PubMed PMID: 32716488](https://pubmed.ncbi.nlm.nih.gov/32716488/?utm_source=chatgpt.com)
36. Pontigo-Loyola, A. P., Mendoza-Rodriguez, M., de la Rosa-Santillana, R., Rivera-Pacheco, M. G., Islas-Granillo, H., Casanova-Rosado, J. F., Márquez-Corona, M. L., Navarrete-Hernández, J. J., Medina-Solís, C. E., & Manton, D. J. (2024). Control of dental caries in children and adolescents using fluoride: An overview of community-level fluoridation methods. Pediatric Reports, 16(2), 243–253. [PubMed PMID: 38651460](https://pubmed.ncbi.nlm.nih.gov/38651460/?utm_source=chatgpt.com)
37. Patel, B., Patrick, A., & Dyer, T. A. (2025). The ethics of community water fluoridation: Part 1—An overview of public health ethics. British Dental Journal, 238(5), 311–315. [PubMed PMID: 40087432](https://pubmed.ncbi.nlm.nih.gov/40087432/?utm_source=chatgpt.com)
38. Patel, B., & Dyer, T. A. (2025). The ethics of community water fluoridation: Part 2—How has the ethics of community water fluoridation been appraised in the literature? A scoping review. British Dental Journal, 238(5), 336–343. [PubMed PMID: 40087447](https://pubmed.ncbi.nlm.nih.gov/40087447/?utm_source=chatgpt.com)
